# Supplementary material for: A data management system for precision medicine
Source: PLOS Digit Health. 2025 Jan 9;4(1):e0000464. doi: 10.1371/journal.pdig.0000464 (PMC11717228; doi:10.1371/journal.pdig.0000464)
Supplement: Attachment — Submitted filename: Response to Reviewers-v5.docx [file pdig.0000464.s001.docx]

PDIG-D-24-00057
A data management system for precision medicine
A rebuttal letter that responds to each point raised by the editor and reviewer(s).

New response to editor

Dear Editor,

Thank you for your additional questions and comments to our “A data management system for precision medicine” and providing extra time for corrections to our manuscript. We would like to refer to Response to Reviewers-v4 for our answers to your previous comments, this mail deals with version 5 and contains updates after your last mail only. The mail below highlights the changes made and answers your questions. All files have been uploaded to the editorial manager as v5.

1. Financial disclosures of all authors have been added to the manuscript.
   - **Financial disclosure:** The authors of this paper acknowledge funding from various sources. JJLJ, IV, IB, SH, FE, CS, SNV, MJ, CS & MH acknowledge funding from the EU framework project AIDPATH (grant agreement number 101016909). VAMCB, HJAO & RCV acknowledge funding from ZonMw (grant number 10140302110018), the Netherlands. AHMZ, LDB, & YW acknowledge funding from the PPP Allowance by Health Holland, Top Sector Life Sciences & Health (LSHM20104; LSHM20068), the Netherlands, and by Novartis. JC (institute): speakers’ bureau of Astellas, research funding Takeda, DC-one, Genentech, Janssen, Novartis and Merus, royalties from BD biosciences and Navigate. LLN, PG, JT & AV have no financial disclosures to declare. The funders had no role in study design, data collection and analysis, decision to publish, or preparation of the manuscript.
2. The data availability statement was updated to the institutional mail address.
   - **Data availability statement:** All data used for conclusion data are in the manuscript and/or supporting information files. This paper also describes the collection and use of various datasets of which the scientific conclusions, where applicable the paper refers to these papers. GDPR restricts publication of these pseudonymous data. Some data is only collected in this paper, and therefore not used for any scientific conclusions in this paper. Access to various datasets can be requested through [info-nl@ortec.com](mailto:info-nl@ortec.com).
3. Figure 1 was converted a .tif file. All .tif files are less than 1 MB.
4. All images were drawn by the authors, without using any copyrighted images or clip arts. Figure 1 was drawn by Steven van Dijk using Drawio and figures 2, 3, 4, and 6 were drawn by John Jacobs using Powerpoint.
5. Referring to unpublished data in lines 412 and 423 has been removed.

I hope this update further addresses all questions regarding the manuscript sufficiently. Should, however, anything still be unclear, incorrectly, or incompletely addressed please don’t hesitate to contact me.

Kind regards,

John Jacobs

John.Jacobs@ortec.com
