# Supplementary material for: A data management system for precision medicine
Source: PLOS Digit Health. 2025 Jan 9;4(1):e0000464. doi: 10.1371/journal.pdig.0000464 (PMC11717228; doi:10.1371/journal.pdig.0000464)
Supplement: Attachment — Submitted filename: Response to Reviewers-v6.docx [file pdig.0000464.s002.docx]

PDIG-D-24-00057R1
A data management system for precision medicine
A rebuttal letter that responds to each point raised by the editor and reviewer(s).

Response to Reviewers

Dear Editor,

Thank you for reviewing our manuscript “A data management system for precision medicine” and considering it for publication in PLOS Digital Health. We are grateful for the comments by the reviewer and editors, which we have used to improve the manuscript. Your points have been addressed as indicated below:

1. The reference list has been reviewed, which has led to minimal text editing.
   - In some papers the last page number was added (refs 1, 9).
   - Some papers had article numbers instead of page numbers (p.), this was corrected accordingly (refs 6,20,21, 25, 27, 32, 33, 35, 47, 50, 51, 62, 65, 66, 69, 70, 71 75, 76, 77, 78).
   - A typo (space) in the link for ref 44 was corrected.
   - The aberrant issue number in ref 47 was removed.
   - Reference 52 has been published and the article number is given.
2. The competing interest statement is completed for all authors.
   - **Conflicts of interest:** We have read the journal's policy and the authors of this manuscript have the following competing interests: JJLJ, IB, IV, LBR, & SD are employees of ORTEC B.V. which has a commercial interest in LogiqSuite. AV, LDB, VAMCB, JC, FE, PG, SH, MH, MJ, AHMZ, SNV, LLN, QAR, CS, JT, HJAO, RCV & YW do not have any conflicts of interest to declare.
3. All images have been provided in *.tif format. All images have been removed from the manuscript text.
   - The figures have been converted at the Preflight Analysis and Conversion Engine (PACE) digital diagnostic tool for validity check.
   - All images were drawn by the authors, without using any copyrighted images or clip arts. Figure 1 was drawn by Steven van Dijk using Drawio and figures 2, 3, 4, and 6 were drawn by John Jacobs using Powerpoint.
4. Financial disclosures of all authors have been added to the manuscript.
   - **Financial disclosure:** The authors of this paper acknowledge funding from various sources. JJLJ, IV, IB, SH, FE, CS, SNV, MJ, CS & MH acknowledge funding from the EU framework project AIDPATH (grant agreement number 101016909). VAMCB, HJAO & RCV acknowledge funding from ZonMw (grant number 10140302110018), the Netherlands. AHMZ, LDB, & YW acknowledge funding from the PPP Allowance by Health Holland, Top Sector Life Sciences & Health (LSHM20104; LSHM20068), the Netherlands, and by Novartis. JC (institute): speakers’ bureau of Astellas, research funding Takeda, DC-one, Genentech, Janssen, Novartis and Merus, royalties from BD biosciences and Navigate. LLN, PG, JT & AV have no financial disclosures to declare. The funders had no role in study design, data collection and analysis, decision to publish, or preparation of the manuscript.
5. The editor and reviewers requested a data availability statement without the corresponding author as a fall-back scenario. Often the ethical board would be a suitable central place for data requests, but this paper describes seven research lines from 5 different multicentred international consortia, implying different ethical requests at different institutes. Providing an extensive list of ethical boards would not facilitate easy data availability request. Therefore we’ve contacted the editorial board to find a more stream-lined solution. Early email correspondence with dr Jessica Thomas at 17 & 18.04.2024 suggested that a link to ORTEC’s site would be an improvement to contacting multiple ethical boards. Later, correspondence with dr Shaira Joy Petancio (26.04 & 09.05.2024) provided progressive insights, that a generic company email address would suffice. This has been added to the manuscript.
   - **Data availability statement:** All data used for conclusion data are in the manuscript and/or supporting information files. This paper also describes the collection and use of various datasets of which the scientific conclusions, where applicable the paper refers to these papers. GDPR restricts publication of these pseudonymous data. Some data is only collected in this paper, and therefore not used for any scientific conclusions in this paper. Access to various datasets can be requested through the program leads or via the corresponding author.
6. Some minor textual changes have been made:
   - Minor corrections in spelling author names of Sergio Navarro-Velázquez & Qasim A. Rafiq. Completed name of Foundation for Biomedical Research-IDIBAPS, Barcelona, Spain. Corrected department of affiliation from author PG.
   - Textual correction in line 174-175. It now reads The Sciencrew platform allows publishing data dictionaries for integration with LogiqSuite.”
   - Minor corrections of typos in line 18, 215, 261,
   - 441, 646, 722.
   - Minor corrections in lines 544 to 548. **Author contributions:** Authors contributed on *medical data science* (JJLJ, IB, IV, & LBR), *LogiqSuite design* (JJLJ, IB, IV, LBR, & SvD) and the use-cases *AML-MRD* (JC, LLN, JT, PG, JJLJ, IB, IV, & LBR), *P4O2* (AHMZ, LDB, IB, IV, JJLJ, LBR, & YW), *AID PATH database* (JJLJ, IV, IB, SH, FE, CS, SNV, MJ, & MH), *AIDPATH AI,* (JJLJ, IV, AV, IB, SNV, MJ, CS, & MH), *AML MRD AI* (AV, JJLJ, IB, IV, JC, LLN, & JT), *Stroke Triage App* (IB, & JJLJ), *Prospera* (VAMCB, HJAO, RCV, JJLJ, IB, & IV), and *Idea & initial draft writing* (JJLJ). All authors contributed to the paper and agreed with its contents.
7. Besides this 'Response to Reviewers' also the marked-up copy titled 'Revised Manuscript with Track Changes' and an unmarked version of the revised paper labelled ‘Manuscript’ have been uploaded.

We hope we’ve addresses all questions regarding the manuscript sufficiently. Should, however, anything still be unclear, incorrectly, or incompletely addressed please don’t hesitate to contact me.

Kind regards,

On behalf of the authors,

John Jacobs

John.Jacobs@ortec.com
